# Effects of cognitive-motor interventions on serum BDNF levels in adults and older population post-stroke: a systematic review and meta-analysis of randomized controlled trials

**DOI:** 10.3389/fphar.2026.1835358

**Published:** 2026-05-07

**Authors:** Edgar Vásquez-Carrasco, Valentina Albornoz-Gómez, Emilia Cerda-González, Constanza González-Guerra, Josefa Salazar-Díaz, Jordan Hernandez-Martinez, Cristian Sandoval-Vásquez, Pablo Valdés-Badilla

**Affiliations:** 1 School of Occupational Therapy, Faculty of Psychology, Universidad de Talca, Talca, Chile; 2 Centro de Investigación en Ciencias Cognitivas, Facultad de Psicología, Universidad de Talca, Talca, Chile; 3 Vitalis Longevity Center, Universidad de Talca, Talca, Chile; 4 Department of Physical Activity Sciences, Universidad de Los Lagos, Osorno, Chile; 5 Department of Education, Faculty of Humanities, Universidad de La Serena, La Serena, Chile; 6 Carrera de Terapia Ocupacional, Facultad de Ciencias de la Salud, Universidad Autónoma de Chile, Temuco, Chile; 7 Departamento de Medicina Interna, Facultad de Medicina, Universidad de La Frontera, Temuco, Chile; 8 Department of Physical Activity Sciences, Faculty of Education Sciences, Universidad Católica del Maule, Talca, Chile; 9 Sports Coach Career, Faculty of Life Sciences, Universidad Viña del Mar, Viña del Mar, Chile

**Keywords:** aged, brain vascular disorders, disability, dual task, rehabilitation

## Abstract

**Introduction:**

Cognitive–motor interventions have emerged as promising rehabilitation strategies to enhance neuroplasticity following stroke; however, their effects on circulating brain-derived neurotrophic factor (BDNF) remain incompletely understood. This systematic review and meta-analysis aimed to quantify the effects of cognitive–motor interventions on serum BDNF levels in adults and older adults after stroke.

**Methods:**

A comprehensive literature search of MEDLINE/PubMed, Scopus, Cochrane, Web of Science (Core Collection), EBSCOhost, CINAHL, and ProQuest was conducted through December 2025. Randomized controlled trials evaluating cognitive–motor interventions and reporting serum brain-derived neurotrophic factor outcomes in post-stroke populations were included. Methodological quality and certainty of evidence were assessed according to PRISMA 2020 guidelines, the Oxford Centre for Evidence-Based Medicine framework, the RoB two tool, and GRADEpro. The review protocol was registered in PROSPERO (CRD420251140852).

**Results:**

Nine randomized controlled trials involving 457 participants met the inclusion criteria. Meta-analysis demonstrated that cognitive–motor interventions elicited a statistically significant increase in serum BDNF levels compared with control conditions (Hedges’ g = 2.51; 95% CI: 0.97 to 4.06; p = 0.001; I^2^ = 92%), indicating a large overall effect.

**Conclusion:**

These findings suggest that cognitive-motor interventions may increase circulating BDNF levels after stroke, supporting their potential role in promoting neuroplastic mechanisms during rehabilitation; however, the very high heterogeneity across studies warrants cautious interpretation.

**Systematic Review Registration:**

https://www.crd.york.ac.uk/PROSPERO/view/CRD420251140852 identifer, PROSPERO 2025 CRD420251140852.

## 1 Introduction

Stroke, a disease with multiple origins influenced by genetic and environmental factors, ranks among the primary causes of disability and mortality globally (Feigin et al., 2017). The significance is demonstrated by a 70% increase in stroke incidence and an 85% rise in prevalent instances in recent years (Du et al., 2023). Stroke presently ranks as the second major cause of mortality and the third leading cause of death and disability globally, with an estimated 101 million prevalent cases worldwide. It is responsible for approximately 6.55 million fatalities and 143 million disability-adjusted life years (DALYs) lost, indicating its significant impact on the worldwide disability burden (Zhang et al., 2023). Furthermore, the scale of the issue is significant, as stroke is among the primary causes of disability in affluent nations, impacting one in six persons (Nandan et al., 2023). Conversely, the majority of stroke survivors encounter cognitive, motor, sensory, and emotional deficits that restrict their engagement in daily activities and impede their reintegration into society and the workforce (Wu et al., 2021). In this context, additional study is necessary, as both global population expansion and demographic shifts linked to heightened life expectancy may exacerbate the burden of stroke (Drescher et al., 2022).

Due to its high incidence, rehabilitation is crucial for the recovery of stroke survivors, and numerous studies have examined various exercises and treatment approaches (Eun et al., 2022). In this regard, electrical stimulation has been shown to be a therapeutic method capable of reducing post-stroke gait dysfunctions (Da Cunha et al., 2021). Similarly, virtual reality (VR) therapy for the upper limb may be effective in improving motor rehabilitation outcomes (Chen et al., 2022). Stroke patients should receive rehabilitation delivered by multidisciplinary teams, including rehabilitation physicians, nurses, physiotherapists, occupational therapists, speech and language therapists, and social workers (An et al., 2024). Cognitive stimulation combined with physical rehabilitation not only improves motor function but also facilitates greater integration of cognitive skills into activities of daily living (Herrera-Samartín et al., 2024). Cognitive-motor interventions integrate motor execution and cognitive engagement simultaneously during rehabilitation, encompassing modalities such as dual-task training, aerobic exercise combined with cognitive tasks, virtual or augmented reality, exergames, and robotic therapies (Mou and Jiang, 2025; Vásquez-Carrasco et al., 2025a). By incorporating cognitive demands into motor practice, these interventions more closely mirror real-life functional activities and may enhance neuroplastic processes (Vásquez-Carrasco et al., 2025b).

Currently, brain-derived neurotrophic factor (BDNF) is one of the most widely studied neurotrophins, as low BDNF levels have been associated with an increased risk of stroke, larger infarct volume, and poorer long-term functional outcomes (Karantali et al., 2021). Increasing evidence indicates a direct relationship between physical exercise and improved brain function, as strength exercise is one of the key factors that trigger the release of BDNF. From a physiological perspective, this may contribute to enhanced post-stroke neuroplasticity, including synaptic plasticity (Górna and Domaszewska, 2022).

Neuroplasticity refers to the brain’s capacity to rearrange itself by forming new neural connections in reaction to learning, experience, and injury (Drigas and Sidarki, 2024). This phenomena encompasses multiple mechanisms, including synaptic plasticity, dendritic remodeling, and alterations in neural connections, facilitating the brain’s capacity for flexible and ongoing reorganization (Leeb et al., 2011). BDNF serves a crucial function within these processes. This neurotrophin, synthesized in the nervous system, facilitates neuronal survival and proliferation, and is implicated in learning, memory, and activity-dependent neuronal plasticity (Park and Poo, 2013). BDNF has been recognized as a prospective biomarker for post-stroke recovery in humans, and therapies that enhance BDNF levels exhibit therapeutic promise (De Las Heras et al., 2023). Although serum BDNF does not directly quantify central nervous system activity, it serves as a peripheral indicator of activity-dependent plasticity and biological responsiveness to rehabilitation (Chen et al., 2023). Therefore, measuring BDNF can complement functional outcomes and contribute to understanding the underlying mechanisms of recovery after stroke. This systematic review and meta-analysis sought to determine the impact of cognitive-motor treatments on serum BDNF levels in adults and the elderly following a stroke.

## 2 Methods

### 2.1 Protocol and registration

This systematic review and meta-analysis were conducted in accordance with the Cochrane Collaboration guidelines (Higgins et al., 2023) and reported following the PRISMA 2020 statement, including both the checklist and flow diagram (Page et al., 2021). The study protocol was prospectively registered in the PROSPERO (CRD420251140852).

### 2.2 Eligibility criteria

This systematic review and meta-analysis included peer-reviewed original studies, specifically randomized controlled trials (RCTs), with no restrictions on language or publication date up to December 2025. Excluded publications comprised conference abstracts, books and book chapters, editorials, letters to the editor, protocols, reviews, case reports, and non-randomized studies. Study eligibility and selection were guided by the PICOS framework (Population, Intervention, Comparator, Outcomes, and Study design), as detailed in Table 1.

**TABLE 1:** Selection criteria used in the systematic review and meta-analysis.

Category	Inclusion	Exclusion
Population	Studies involving adults aged 18 years or older with a diagnosis of stroke	Studies in which the primary condition was not stroke (e.g., multiple sclerosis, amyotrophic lateral sclerosis, traumatic brain injury), and studies including participants under 18 years of age
Intervention	Studies including a cognitive-motor intervention for 4 weeks or more	Studies including other types of complementary interventions not related to cognitive–motor interventions
Comparison	Interventions with active or inactive control groups	Lack of baseline and/or follow-up data. Absence of control group
Outcomes	Studies that present results of serum BDNF levels in adult and older population post-stroke	Lack of baseline data and/or follow-ups
Study design	Randomized controlled trials, with pre- and post-assessment	Controlled, retrospective, prospective and cross-sectional, non-randomized studies
Level of evidence	1b	2a, 2b, 3a, 3b, 4 and 5

BDNF: Brain-Derived Neurotrophic Factor.

### 2.3 Information search process and database

A comprehensive literature search was conducted across seven electronic databases: MEDLINE/PubMed, Scopus, Cochrane Library, Web of Science (Core Collection), EBSCOhost, CINAHL, and ProQuest. The investigation was conducted from March to November 2025. The U.S. National Library of Medicine’s Medical Subject Headings (MeSH) were utilized alongside pertinent free-text phrases concerning neuroplasticity-based cognitive-motor therapies in adults and the elderly, as well as stroke. The subsequent search technique was implemented: (“Stroke” OR “cerebrovascular accident” OR “cerebral infarct” OR “cerebral infarction” OR “cerebral hemorrhage” OR “ischemic stroke” OR “hemorrhagic stroke” OR “brain ischemia” OR “cerebrovascular disease” OR “cerebrovascular insult” OR “transient ischemic attack” OR “TIA”) AND (“Brain-Derived Neurotrophic Factor” OR “BDNF” OR “BDNF Val66Met” OR “pro-BDNF” OR “mature BDNF” OR “BDNF gene” OR “neurotrophic factor” OR “neurotrophin”) AND (“Aged” OR “Adult” OR “elderly” OR “older adults” OR “older population” OR “senior” OR “middle-aged” OR “geriatric”). See full search strategy in Supplementary Table S1.

### 2.4 Study selection process and data collection

All retrieved records were exported to Mendeley Reference Manager (version 2.116.3, Elsevier, London, United Kingdom), and the study selection process is summarized in the PRISMA flow diagram. Four authors independently conducted the searches and systematically screened titles, abstracts, and full texts. Duplicate records were removed, and no discrepancies were identified at this stage. Potentially eligible articles were subsequently assessed in detail, with exclusions documented according to predefined eligibility criteria. Two authors independently audited the entire selection and data extraction process.

### 2.5 Methodological quality assessment

Methodological quality and level of evidence were evaluated using the Oxford Centre for Evidence-Based Medicine scale (Manterola and Zavando, 2009). Only RCTs classified as level 1b were eligible for inclusion, while studies rated from levels 2a to five were excluded. The included RCTs were downgraded when issues related to risk of bias, consistency, accuracy, precision, or transparency of reporting were identified (Manterola and Zavando, 2009).

### 2.6 Data synthesis

Data from the included studies were extracted using a standardized form in Microsoft Excel (version 2,506; Microsoft Corporation, Redmond, WA, United States), in accordance with Cochrane guidelines (Higgins et al., 2023). Four authors independently performed data extraction and compared their results to ensure accuracy. A fifth author supervised the process. Extracted variables included authors, country, study design, sample size, group sizes (n), mean age (years), type of intervention and control, training volume (frequency, duration, and intensity), outcome assessments, and main outcomes.

### 2.7 Risk of bias

Risk of bias in the included RCTs was evaluated using the Cochrane RoB two tool (Cochrane, 2020). Two reviewers conducted the assessments, which were independently reviewed by another author. Disagreements were resolved through discussion until consensus was reached.

### 2.8 Meta-analysis measures

This study employed a meta-analytic approach, with the detailed methodology prospectively registered in PROSPERO (CRD420251140852). Standardized mean differences (SMDs) were calculated to quantify between-group differences in RCTs for each comparison using Review Manager software (RevMan 5.4, Cochrane Collaboration, London, UK). Statistical significance was set at p < 0.05 (Verhagen et al., 1998). A random-effects method was applied to pool SMDs and mean differences for neuroplasticity outcomes measured through serum BDNF, comparing experimental and control groups before and after the intervention (Higgins and Green, 2008). This model accounts for between-study variability in true intervention effects, reflecting differences in intervention type, duration, and population characteristics. Meta-analyses were conducted when at least three studies reported comparable data (Davey et al., 2011). Heterogeneity was assessed using Cochran’s Q test and the I^2^ statistic, with I^2^ values interpreted as low (<25%), moderate (25%–50%), or high (>50%) inconsistency (Higgins and Green, 2008). In addition, Egger’s regression test was performed to evaluate small-study effects and potential publication bias (Cochrane, 2020). A leave-one-out sensitivity analysis was conducted by sequentially removing one study at a time and recalculating the pooled effect size and heterogeneity estimates to assess the robustness of the overall result and the potential influence of individual trials on the summary estimate.

### 2.9 Certainty of evidence

The certainty of evidence was evaluated utilizing the GRADE framework (Cochrane, 2024) and categorized as high, moderate, low, or very low. All analyses commenced with a high degree of certainty owing to the incorporation of RCTs but were subsequently downgraded due to concerns about risk of bias, consistency, accuracy, precision, transparency, or publication bias. Four reviewers performed separate evaluations, and any conflicts were reconciled through consensus among the same reviewers, in accordance with the GRADE instruction manuals.

## 3 Results

### 3.1 Study selection

The database search identified a total of 4,159 records, of which 578 were removed as duplicates. Of the remaining 3,581 records, 3,524 were excluded after title and abstract screening for relevance (2,277 based on title and 1,247 based on abstract). Following full-text assessment of 57 articles, 48 studies were excluded for not meeting the predefined inclusion criteria: 14 due to incomplete intervention focus, 18 because they addressed unrelated topics, and 16 because they did not meet the required study design. Ultimately, nine studies were included in the systematic review and meta-analysis (Ajum et al., 2019; Huang et al., 2022; Kim et al., 2019; Koroleva et al., 2020; Petrova et al., 2023; Ploughman et al., 2019; Usova et al., 2025; Wang et al., 2021; Zhao et al., 2022).

### 3.2 Methodological quality

**FIGURE 1:**
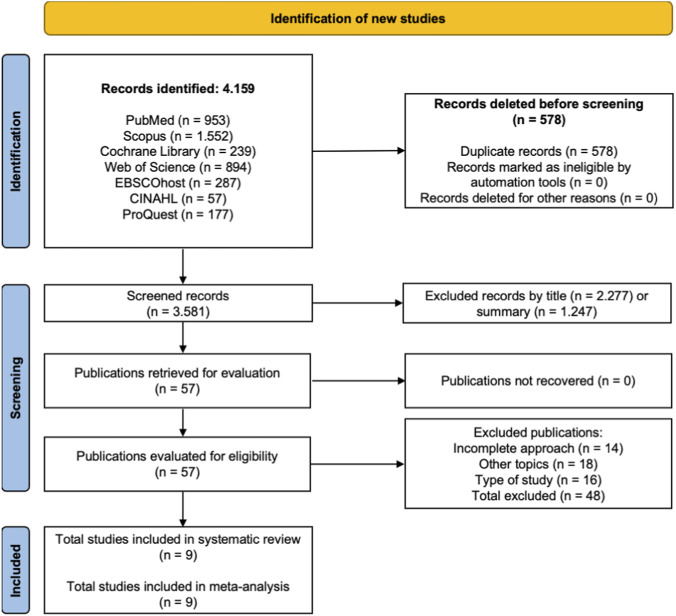
PRISMA 2020 flow diagram describing the identification, screening, and inclusion of studies (Page et al., 2021).

The methodological quality of the studies included in this systematic review was considered high. All selected studies were RCTs, representing the highest level of evidence (level 1b) according to the Oxford Centre for Evidence-Based Medicine. These RCTs were conducted across multiple countries, including Pakistan, Taiwan, South Korea, Russia, Canada, and China, and examined the effects of cognitive–motor interventions on neuroplasticity-related factors, specifically serum BDNF, in adult and older population stroke survivors (Ajum et al., 2019; Huang et al., 2022; Kim et al., 2019; Koroleva et al., 2020; Petrova et al., 2023; Ploughman et al., 2019; Usova et al., 2025; Wang et al., 2021; Zhao et al., 2022). Figure 1 shows PRISMA flowchart.

### 3.3 Risk of bias

**FIGURE 2:**
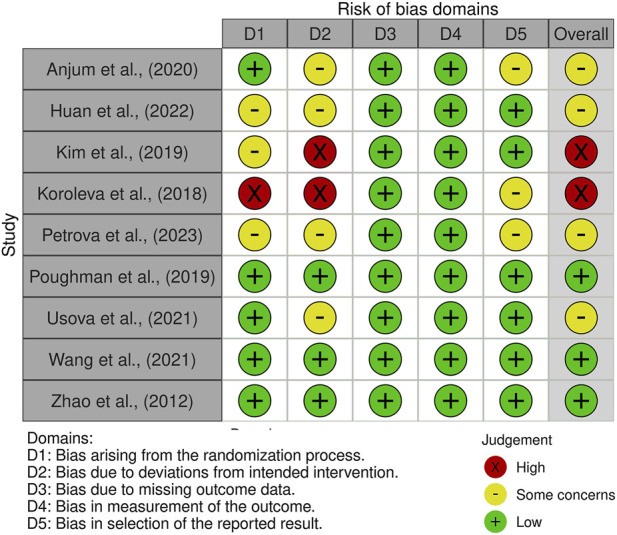
Summary of the risk of bias assessments across the included randomized controlled trials.

**FIGURE 3:**
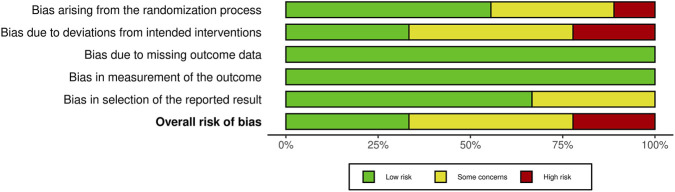
Risk of bias traffic-light plot showing domain-specific judgments for each study.

Three studies were assessed as having a low risk of bias (Ploughman et al., 2019; Wang et al., 2021; Zhao et al., 2022). Four studies raised some concerns regarding risk of bias (Ajum et al., 2019; Huang et al., 2022; Petrova et al., 2023; Usova et al., 2025). Two studies were judged to have a high risk of bias (Kim et al., 2019; Koroleva et al., 2020). Only two studies were rated as having a high risk of bias, supporting the overall reliability of the remaining findings. Figures 2, 3 summarize the risk-of-bias assessments. Supplementary Figure S1 shows forest plot of changes in serum BDNF in people with stroke participating in cognitive motor intervention, while Supplementary Figure S2 shows heterogeneity analysis and Supplementary Table S2 shows leave-one-out sensitivity analysis of pooled effects on serum BDNF levels.

### 3.4 Sample characteristics

All nine RCTs included cognitive-motor therapies aimed at neuroplasticity-related aspects in adults and older individuals following a stroke. The principal neuroplasticity outcome evaluated in most research was serum BDNF (Ajum et al., 2019; Huang et al., 2022; Kim et al., 2019; Koroleva et al., 2020; Petrova et al., 2023; Ploughman et al., 2019; Usova et al., 2025; Wang et al., 2021; Zhao et al., 2022). The duration of interventions varied from 4 to 24 weeks, predominantly consisting of 4-week protocols (Ajum et al., 2019; Usova et al., 2025; Zhao et al., 2022), with one study extending to 12 weeks (Koroleva et al., 2020). Session frequency varied from two to three sessions weekly (Huang et al., 2022) to three sessions weekly (Petrova et al., 2023; Ploughman et al., 2019), and as many as seven sessions weekly (Koroleva et al., 2020); one randomized controlled trial did not disclose session frequency (Wang et al., 2021). Session time varied from 7.5 min (Usova et al., 2025) to 50-70 min (Ploughman et al., 2019). Mean group sizes varied between 18 and 100 individuals, with an average age of 52 years (Table 2).

**TABLE 2:** Selected studies on the effects of cognitive–motor interventions on neuroplasticity-related factors in adult and older population post stroke.

Authors	Country	Study design	Initial sample	Groups (n)	Mean age (years)	Type of group intervention	Training volume			Assessment	Main outcomes
							Weeks	Frequency (sessions/Weeks)	Session duration (min)		
Ajum et al. (2019)	PK	RCT	Stroke	EG: 20 CG: 20	45	EG: Intervention using nintendo Wii for upper limb CG: Traditional physiotherapy group	4	4	20	Serum BDNF levels	↑ Serum BDNF levels (p<0.001)
Huang et al. (2022)	TW	RCT	Stroke	EG: 15 CG: 15	54.2	EG: Visual refraction therapy group CG: Continuous occupational therapy group	16	2-3	60	Serum BDNF levels	↑ Serum BDNF levels (p=0.023)
Kim et al. (2019)	KR	RCT	Stroke	EG: 9 CG: 9	57.5	EG: High-intensity aerobic exercise plus dual-task training group CG: Low intensity aerobic exercise plus dual-task training group	6	5	The exercise time and speed were calculated using formulas published by the ACSM	Serum BDNF levels	↑ Serum BDNF levels (p<0.001)
Koroleva et al. (2020)	RU	RCT	Stroke	EG: 14 CG: 15	49	EG: AR-based rehabilitation within the early recovery period CG: Usual care	12	7	20 min. The rest time between tasks within the domain, at the request of the patient, was 1–2 min; that between the four motor tasks was 5 min	Serum BDNF levels	↑ Serum BDNF levels (p<0.05)
Petrova et al. (2023)	RU	RCT	Stroke	EG: 46 CG: 44	58	EG: rehabilitation using a sensory glove combined with virtual reality CG: received individualized physiotherapy	5	3 sessions/week	30	Serum BDNF levels	↑ Serum BDNF levels (p=0.042)
Ploughman et al. (2019)	CA	Two-site RCT	Chronic Stroke	Aerobic + COG: 15 Aerobic + Games: 15 Activity + COG: 15 Activity + Games: 15	63.4	Physical: Aerobic treadmill training OR therapeutic Activity Cognitive: Adaptive dual n-back working memory training (COG) OR computer puzzle games (Control)	10	3 sessions/week	50–70 min (20–30 min physical +20–30 min cognitive)	Serum BDNF levels	No significant change in BDNF with any intervention
Usova et al. (2025)	RU	RCT	Stroke	EG: 59 CG: 38	58.2	EG: Receives rehabilitation without the use of VR CG: Usual care	4	5 – 10 sessions	2.5 min x 3 times	Serum BDNF levels	↑ Serum BDNF levels (p<0.001)
Wang et al. (2021)	CN	RCT	Stroke	EG: 50 CG: 50	65.3	EG: Rehabilitation training based on routine drug therapy CG: Routine medication	24	NR	NR	Serum BDNF levels	↑ Serum BDNF levels (p=0.002)
Zhao et al. (2022)	CN	RCT	Stroke	EG: 14 CG: 14	52.4	EG: BCI-controlled robot group conventional PT and medical treatments CG: Conventional PT and medical treatments	4	6	30	Serum BDNF levels	↑ Serum BDNF levels (p<0.001)

Interventions varied widely in content and intensity, which may explain the high heterogeneity observed; ACSM: american college of sports medicine; AR: augmented reality; BCI: Brain–Computer Interface; BDNF: Brain-Derived Neurotrophic Factor; CA: canada; CG: control group; CN: china; COG: cognitive training; EG: experimental group; KR: corea del sur; PK: pakistan; NR: not reported; PT: physiotherapy; RCT: randomized controlled trial; RU: russia; TW: taiwan; VR: virtual reality.

### 3.5 Brain-derived neurotrophic factor

**FIGURE 4:**
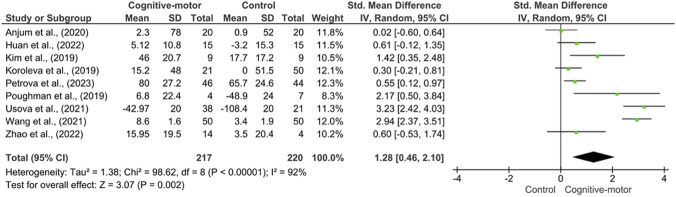
Forest plot showing changes in BDNF levels in adults and older population with stroke compared with control groups. Effect sizes are presented as Hedges’ g with 95% confidence intervals, and the size of each square reflects the relative statistical weight of the corresponding study.

When compared with control conditions, cognitive-motor interventions were associated with a statistically significant increase in serum BDNF levels (Hedges’ g = 2.51; 95% CI: 0.97 to 4.06; p = 0.001). However, heterogeneity was very high (I^2^ = 92%, p < 0.001), indicating marked between-study variability. A leave-one-out sensitivity analysis showed that the pooled effect remained statistically significant after sequential exclusion of each individual study, with effect sizes ranging from 1.82 to 2.84, while heterogeneity remained consistently high across all iterations (I^2^ range: 95.1%-97.3%). These findings suggest that the overall effect was not driven by a single study, although the persistently high heterogeneity indicates that the pooled estimate should be interpreted with caution. Egger’s regression test did not show statistically significant evidence of small-study effects (p = 0.112), although this result should be interpreted cautiously given the limited number of included studies (Figure 4).

### 3.6 Certainty of evidence

According to the GRADEpro assessment, the overall certainty of the evidence for the effect of cognitive-motor interventions on serum BDNF levels was rated as moderate. This judgment was based on nine randomized controlled trials and was downgraded due to concerns related to risk of bias and inconsistency, with inconsistency primarily explained by the very high heterogeneity detected in the meta-analysis. No serious concerns were identified for indirectness or imprecision. Overall, the evidence suggests that cognitive-motor interventions may increase serum BDNF levels after stroke; however, the substantial heterogeneity across studies warrants cautious interpretation, and further high-quality randomized controlled trials are needed to confirm these findings (Table 3).

**TABLE 3:** Evaluation of methodological quality using the GRADEpro tool.

Certainty assessment							Number of patients		Effect		Certainty	Importance
Number of studies	Study design	Risk of bias	Inconsistency	Indirect evidence	Imprecision	Other considerations	[Intervention]	[Comparison]	Relative (95% CI)	Absolute (95% CI)	​	​
BDNF												
9	RCT	Serious^a^	Serious	Not serious	Not serious	None	256/512 (50.0%)	256/512 (50.0%)	Not serious	​	Moderate	IMPORTANT

^a^ Some concerns; CI: confidence interval; RCT: randomized trials.

### 3.7 Effects adverse and adherence

The studies included in this systematic review and meta-analysis reported a 93% adherence rate and no adverse effects, indicating that the interventions were feasible and well tolerated by adult and older population post stroke. These findings highlight their potential for broader application in similar populations.

## 4 Discussion

### 4.1 Brain-derived neurotrophic factor

This systematic review aimed to integrate and critically evaluate the available evidence on the effects of cognitive-motor interventions on serum BDNF levels in adults and older individuals after stroke. By focusing on randomized controlled trials, the review enabled a comprehensive analysis of intervention characteristics, outcome measures, and methodological quality, with particular emphasis on BDNF as an indirect marker of neuroplasticity.

The meta-analysis demonstrated a significant effect of cognitive-motor interventions on serum BDNF levels, showing a marked increase compared with control conditions. However, this finding should be interpreted with caution given the very high heterogeneity across studies, which likely reflects important clinical and methodological differences among the included trials. These findings are consistent with previous evidence indicating that structured physical exercise, particularly at moderate to vigorous intensities, is associated with significant elevations in circulating BDNF. For example, Dinoff et al. (2017) reported that aerobic exercise interventions significantly increased serum BDNF levels (p < 0.0001), while El-Tamawy et al. (2014) observed a substantial post-intervention rise in BDNF in individuals following stroke. These results suggest that exercise-based interventions may positively modulate activity-dependent neuroplasticity mechanisms that support neural reorganization and functional recovery after stroke.

Supporting this interpretation, a meta-analysis of 29 studies involving 1,111 participants found that acute exercise elicited a moderate increase in BDNF levels (Hedges’ g = 0.46; p < 0.001) (Szuhany et al., 2015). In contrast, Maguire et al. (2023) reported no significant changes in BDNF following a 12-week moderate-intensity exercise program. Rather than contradicting existing evidence, these null findings may reflect methodological constraints, such as limited sample size, which reduce statistical power and increase the risk of false-negative results (Button et al., 2013). Moreover, insufficient exercise intensity may fail to trigger the neurobiological threshold required for detectable BDNF modulation, highlighting the critical role of dosage and training intensity in post-stroke rehabilitation (Boyne et al., 2019).

The clinical relevance of these findings is further reinforced by experimental and human evidence showing that physical activity and enriched environments increase BDNF expression in key brain regions, including the hippocampus, thereby promoting neurogenesis, synaptic plasticity, learning, and memory (Colucci-D’Amato et al., 2020). Beyond its role in neuroplasticity, BDNF is also involved in the regulation of dopaminergic and serotonergic systems, linking it to motivation, reward processing, and behavioural engagement. This interaction may have important clinical implications, as enhanced motivation and adherence are critical determinants of the effectiveness of cognitive-motor rehabilitation programs in stroke populations (Vásquez-Carrasco et al., 2024). Future research should prioritize well-powered RCTs designed to determine the optimal parameters of cognitive-motor interventions, including exercise intensity, training volume, frequency, and progression, to maximize BDNF-related neuroplastic responses after stroke.

### 4.2 Limitations and strengths

This study has several limitations. First, the very high heterogeneity observed across studies reduces confidence in the pooled estimate and limits the generalizability of the findings. This variability likely reflects clinical and methodological differences among trials, including intervention characteristics, comparator conditions, and participant profiles. Second, the diversity of cognitive-motor interventions made it difficult to isolate the specific components associated with changes in serum BDNF levels. Third, although all included studies were randomized controlled trials, several raised concerns regarding risk of bias. Finally, most studies had relatively small sample sizes, which may have reduced statistical power.

Despite these limitations, this review also has important strengths. It was prospectively registered in PROSPERO, which enhanced transparency and reduced the risk of selective reporting. Established methodological frameworks were applied throughout, including PRISMA 2020, RoB 2, GRADE, and the Oxford Centre for Evidence-Based Medicine framework. In addition, clear PICOS-based eligibility criteria were used, and only randomized controlled trials classified as level 1b evidence were included, strengthening the methodological rigor of the review.

### 4.3 Practical applications

Scientific evidence indicates that physical exercise induces a cascade of cellular processes that promote brain plasticity. In this context, BDNF, a neurotrophins closely associated with neuroplasticity, can be upregulated because of physical exercise. Therefore, it is relevant to examine the effects of therapeutic exercise on neuroplasticity and/or peripheral BDNF levels in adult neurological conditions, such as stroke. In settings such as research centers or specialized clinical facilities, measuring serum BDNF levels before and after an intervention may be useful for monitoring biological changes and potentially adjusting therapeutic protocols. Finally, stroke is one of the leading acute neurological events worldwide and is associated with a substantial psychosocial and economic burden. Following neuronal injury, the organism initiates an adaptive and reorganizational process known as neuroplasticity. Among the multiple factors involved in this process, BDNF stands out as a key neurotrophins coordinating neuroplastic responses after various neurological disorders, including stroke (Mojtabavi et al., 2022).

### 4.4 Clinical applications

The elevation of BDNF as an indicator of brain plasticity after physical exercise or cognitive-motor therapies signifies a physiological mechanism that reinforces the theoretical basis of post-stroke recovery. Furthermore, Mou and Jiang (2025) reported that dual-task and cognitive-motor training programs significantly enhance gait, lower-limb motor function, cognitive performance, mental health, and activities of daily living in stroke patients, underscoring the necessity for the development of integrated, evidence-based clinical interventions. In this context, healthcare professionals can design rehabilitation programs that integrate moderate-to-high intensity aerobic exercise, cognitive tasks, and functional challenges customized to the individual’s condition, with the objective of enhancing neuroplasticity, particularly *via* BDNF modulation, and thus maximizing functional recovery. These tactics provide a more thorough, evidence-based rehabilitation method that concurrently addresses functional and cognitive outcomes post-stroke. Although increased serum BDNF may reflect activity-dependent neuroplastic responses, its relationship with meaningful clinical improvement remains to be more clearly established in post-stroke rehabilitation.

### 4.5 Epidemiological applications

The implementation of therapeutic exercise programs aimed at enhancing neuroplasticity mechanisms and, consequently, increasing BDNF levels has significant epidemiological relevance for the prevention of disability and the optimization of quality of life in individuals who have experienced a stroke. This relevance stems from the fact that stroke remains one of the leading causes of acquired disability worldwide, characterized by high prevalence and a growing trend in recurrence rates, resulting in a substantial healthcare, social, and economic burden (GBD, 2021 Stroke Risk Factor Collaborators, 2024).

Furthermore, the application of interventions grounded in scientific evidence contributes to reducing functional dependence, promoting social reintegration, and decreasing the costs associated with long-term care. These benefits translate into an increased healthy life expectancy and improved public health indicators by integrating biological dimensions, such as increased BDNF levels and brain plasticity, clinical aspects of functional recovery, and population-level outcomes, including reduced disease burden. Collectively, these effects support a healthcare model centered on prevention and functional independence.

## 5 Conclusion

The available evidence suggests that cognitive-motor interventions may increase serum BDNF levels in adults and older individuals after stroke. However, the overall certainty of the evidence was moderate, and the very high heterogeneity across studies warrants cautious interpretation of the pooled effect. These findings support the potential of integrated cognitive-motor rehabilitation strategies to promote neuroplasticity after stroke, although further high-quality and methodologically consistent randomized controlled trials are needed to confirm these effects and clarify their clinical relevance.

## Data Availability

The original contributions presented in the study are included in the article/Supplementary Material, further inquiries can be directed to the corresponding authors.
