# Supplementary material for: Effects of cognitive-motor interventions on serum BDNF levels in adults and older population post-stroke: a systematic review and meta-analysis of randomized controlled trials
Source: Front Pharmacol. 2026 May 7;17:1835358. doi: 10.3389/fphar.2026.1835358 (PMC13190536; doi:10.3389/fphar.2026.1835358)
Supplement: Supplementary file 1 [file Supplementaryfile1.docx]

Supplementary Material

# Supplementary Figures and Tables

## Supplementary Figures

**Supplementary Figure 1.** Forest plot of changes in serum BDNF in people with Stroke participating in cognitive motor intervention compared with people with Stroke assigned as controls. Values shown are effect sizes (Hedges' g) with 95% confidence intervals (CI). The size of the squares plotted reflects the statistical weight of each study.

**Supplementary Figure 2.** Funnel plot for heterogeneity analysis.

## Supplementary Tables

# Supplementary Table 1. Search strategy

| **Database** | **Search strategy** |
| --- | --- |
| **MEDLINE/PubMed** | (("Stroke"[Mesh] OR stroke[tiab] OR "cerebrovascular accident"[tiab] OR "cerebral infarct"[tiab] OR "cerebral infarction"[tiab] OR "cerebral hemorrhage"[tiab] OR "ischemic stroke"[tiab] OR "haemorrhagic stroke"[tiab] OR "hemorrhagic stroke"[tiab] OR "brain ischemia"[tiab] OR "brain ischaemia"[tiab] OR "cerebrovascular disease"[tiab] OR "cerebrovascular insult"[tiab] OR "transient ischemic attack"[tiab] OR "transient ischaemic attack"[tiab] OR TIA[tiab])) AND (("Brain-Derived Neurotrophic Factor"[Mesh] OR "brain-derived neurotrophic factor"[tiab] OR BDNF[tiab] OR "BDNF Val66Met"[tiab] OR "pro-BDNF"[tiab] OR "mature BDNF"[tiab] OR "BDNF gene"[tiab] OR "neurotrophic factor"[tiab] OR neurotrophin[tiab] OR neurotrophins[tiab])) AND (("Adult"[Mesh] OR "Aged"[Mesh] OR adult[tiab] OR adults[tiab] OR aged[tiab] OR elderly[tiab] OR "older adult*"[tiab] OR "older people"[tiab] OR "older population"[tiab] OR senior[tiab] OR seniors[tiab] OR "middle-aged"[tiab] OR geriatric[tiab] OR geriatrics[tiab])) |
| **Scopus** | TITLE-ABS-KEY(("stroke" OR "cerebrovascular accident" OR "cerebral infarct" OR "cerebral infarction" OR "cerebral hemorrhage" OR "ischemic stroke" OR "haemorrhagic stroke" OR "hemorrhagic stroke" OR "brain ischemia" OR "brain ischaemia" OR "cerebrovascular disease" OR "cerebrovascular insult" OR "transient ischemic attack" OR "transient ischaemic attack" OR TIA) AND ("brain-derived neurotrophic factor" OR BDNF OR "BDNF Val66Met" OR "pro-BDNF" OR "mature BDNF" OR "BDNF gene" OR "neurotrophic factor" OR neurotrophin OR neurotrophins) AND (adult OR adults OR aged OR elderly OR "older adult*" OR "older people" OR "older population" OR senior OR seniors OR "middle-aged" OR geriatric OR geriatrics)) |
| **Cochrane Library** | (MeSH descriptor: [Stroke] explode all trees OR stroke:ti,ab,kw OR "cerebrovascular accident":ti,ab,kw OR "cerebral infarct":ti,ab,kw OR "cerebral infarction":ti,ab,kw OR "cerebral hemorrhage":ti,ab,kw OR "ischemic stroke":ti,ab,kw OR "haemorrhagic stroke":ti,ab,kw OR "hemorrhagic stroke":ti,ab,kw OR "brain ischemia":ti,ab,kw OR "brain ischaemia":ti,ab,kw OR "cerebrovascular disease":ti,ab,kw OR "cerebrovascular insult":ti,ab,kw OR "transient ischemic attack":ti,ab,kw OR "transient ischaemic attack":ti,ab,kw OR TIA:ti,ab,kw) AND (MeSH descriptor: [Brain-Derived Neurotrophic Factor] explode all trees OR "brain-derived neurotrophic factor":ti,ab,kw OR BDNF:ti,ab,kw OR "BDNF Val66Met":ti,ab,kw OR "pro-BDNF":ti,ab,kw OR "mature BDNF":ti,ab,kw OR "BDNF gene":ti,ab,kw OR "neurotrophic factor":ti,ab,kw OR neurotrophin:ti,ab,kw OR neurotrophins:ti,ab,kw) AND (MeSH descriptor: [Adult] explode all trees OR MeSH descriptor: [Aged] explode all trees OR adult:ti,ab,kw OR adults:ti,ab,kw OR aged:ti,ab,kw OR elderly:ti,ab,kw OR "older adult*":ti,ab,kw OR "older people":ti,ab,kw OR "older population":ti,ab,kw OR senior:ti,ab,kw OR seniors:ti,ab,kw OR "middle-aged":ti,ab,kw OR geriatric:ti,ab,kw OR geriatrics:ti,ab,kw) |
| **Web of Science Core Collection** | TS=(("stroke" OR "cerebrovascular accident" OR "cerebral infarct" OR "cerebral infarction" OR "cerebral hemorrhage" OR "ischemic stroke" OR "haemorrhagic stroke" OR "hemorrhagic stroke" OR "brain ischemia" OR "brain ischaemia" OR "cerebrovascular disease" OR "cerebrovascular insult" OR "transient ischemic attack" OR "transient ischaemic attack" OR TIA) AND ("brain-derived neurotrophic factor" OR BDNF OR "BDNF Val66Met" OR "pro-BDNF" OR "mature BDNF" OR "BDNF gene" OR "neurotrophic factor" OR neurotrophin OR neurotrophins) AND (adult OR adults OR aged OR elderly OR "older adult*" OR "older people" OR "older population" OR senior OR seniors OR "middle-aged" OR geriatric OR geriatrics)) |
| **EBSCOhost** | TX (("stroke" OR "cerebrovascular accident" OR "cerebral infarct" OR "cerebral infarction" OR "cerebral hemorrhage" OR "ischemic stroke" OR "haemorrhagic stroke" OR "hemorrhagic stroke" OR "brain ischemia" OR "brain ischaemia" OR "cerebrovascular disease" OR "cerebrovascular insult" OR "transient ischemic attack" OR "transient ischaemic attack" OR TIA) AND ("brain-derived neurotrophic factor" OR BDNF OR "BDNF Val66Met" OR "pro-BDNF" OR "mature BDNF" OR "BDNF gene" OR "neurotrophic factor" OR neurotrophin OR neurotrophins) AND (adult OR adults OR aged OR elderly OR "older adult*" OR "older people" OR "older population" OR senior OR seniors OR "middle-aged" OR geriatric OR geriatrics)) |
| **CINAHL** | ((MH "Stroke+") OR TI stroke OR AB stroke OR TI "cerebrovascular accident" OR AB "cerebrovascular accident" OR TI "cerebral infarct" OR AB "cerebral infarct" OR TI "cerebral infarction" OR AB "cerebral infarction" OR TI "cerebral hemorrhage" OR AB "cerebral hemorrhage" OR TI "ischemic stroke" OR AB "ischemic stroke" OR TI "haemorrhagic stroke" OR AB "haemorrhagic stroke" OR TI "hemorrhagic stroke" OR AB "hemorrhagic stroke" OR TI "brain ischemia" OR AB "brain ischemia" OR TI "brain ischaemia" OR AB "brain ischaemia" OR TI "cerebrovascular disease" OR AB "cerebrovascular disease" OR TI "cerebrovascular insult" OR AB "cerebrovascular insult" OR TI "transient ischemic attack" OR AB "transient ischemic attack" OR TI "transient ischaemic attack" OR AB "transient ischaemic attack" OR TI TIA OR AB TIA) AND ((MH "Brain Derived Neurotrophic Factor") OR TI "brain-derived neurotrophic factor" OR AB "brain-derived neurotrophic factor" OR TI BDNF OR AB BDNF OR TI "BDNF Val66Met" OR AB "BDNF Val66Met" OR TI "pro-BDNF" OR AB "pro-BDNF" OR TI "mature BDNF" OR AB "mature BDNF" OR TI "BDNF gene" OR AB "BDNF gene" OR TI "neurotrophic factor" OR AB "neurotrophic factor" OR TI neurotrophin OR AB neurotrophin OR TI neurotrophins OR AB neurotrophins) AND ((MH "Adults+") OR (MH "Aged+") OR TI adult OR AB adult OR TI adults OR AB adults OR TI aged OR AB aged OR TI elderly OR AB elderly OR TI "older adult*" OR AB "older adult*" OR TI "older people" OR AB "older people" OR TI "older population" OR AB "older population" OR TI senior OR AB senior OR TI seniors OR AB seniors OR TI "middle-aged" OR AB "middle-aged" OR TI geriatric OR AB geriatric OR TI geriatrics OR AB geriatrics)) |
| **ProQuest** | ab,ti(( "stroke" OR "cerebrovascular accident" OR "cerebral infarct" OR "cerebral infarction" OR "cerebral hemorrhage" OR "ischemic stroke" OR "haemorrhagic stroke" OR "hemorrhagic stroke" OR "brain ischemia" OR "brain ischaemia" OR "cerebrovascular disease" OR "cerebrovascular insult" OR "transient ischemic attack" OR "transient ischaemic attack" OR TIA) AND ("brain-derived neurotrophic factor" OR BDNF OR "BDNF Val66Met" OR "pro-BDNF" OR "mature BDNF" OR "BDNF gene" OR "neurotrophic factor" OR neurotrophin OR neurotrophins) AND (adult OR adults OR aged OR elderly OR "older adult*" OR "older people" OR "older population" OR senior OR seniors OR "middle-aged" OR geriatric OR geriatrics)) |

**Supplementary Table 2.** Leave-one-out sensitivity analysis of pooled effects on serum BDNF levels.

| **Study** | **Pooled Hedges’ g** | **95% CI** | **I²** |
| --- | --- | --- | --- |
| None | 2.51 | 0.97 to 4.06 | 92.0% |
| Anjum et al., (2019) | 2.84 | 1.14 to 4.55 | 96.9% |
| Huan et al., (2022) | 2.49 | 0.79 to 4.19 | 97.2% |
| Kim et al., (2019) | 2.80 | 1.10 to 4.50 | 97.2% |
| Koroleva et al, (2018) | 2.80 | 1.03 to 4.56 | 97.0% |
| Petrova et al., (2023) | 2.47 | 0.70 to 4.24 | 97.1% |
| Ploughman et al., (2019) | 2.82 | 1.15 to 4.49 | 97.2% |
| Usova et al., 2025) | 1.96 | 0.49 to 3.42 | 96.5% |
| Wang et al., (2021) | 1.82 | 0.56 to 3.08 | 95.1% |
| Zhao et al., (2022) | 2.66 | 0.91 to 4.42 | 97.3% |
